# Comparative Analysis of Three Types of Therapeutic Offloading Diabetic Shoes With Custom Made Insole on Plantar Pressure Distribution in Severe Diabetic Charcot Foot

**DOI:** 10.33137/cpoj.v7i1.41780

**Published:** 2024-08-17

**Authors:** M Nouman, R Apiputhanayut, T Narungsri, S Tipchatyotin, T Dissaneewate

**Affiliations:** 1 Sirindhorn School of Prosthetics and Orthotics, Faculty of Medicine, Siriraj Hospital, Mahidol University, Bangkok, Thailand.; 2 Department of Rehabilitation Medicine, Faculty of Medicine, Prince of Songkla University, Songkhla, Thailand; 3 Prosthetics and Orthotics Unit, Department of Rehabilitation Medicine, Faculty of Medicine, Prince of Songkla University, Songkhla, Thailand

**Keywords:** Neuroarthropathy, Therapeutic Shoe, Charcot Foot, Diabetic Foot, Plantar Pressure, Custom Made Insole, Orthotics, Orthosis, Gait

## Abstract

**BACKGROUND:**

Charcot foot deformity, a severe complication of diabetes, involves neuropathy and abnormal peak plantar pressure in the midfoot and forefoot. However, orthotic interventions and shoe modifications are used to address the sequelae of Charcot neuroarthropathy, offering different approaches to managing abnormal peak plantar pressure.

**OBJECTIVE:**

To compare the effects of three types of therapeutic offloading diabetic shoes; prefabricated, relasting, and double rocker-modified shoes on peak plantar pressure in the midfoot and forefoot of nonulcerated chronic Charcot foot during walking.

**METHODOLOGY:**

A repeated measure design involved 15 participants (40% males and 60% females) with a mean age of 60.73 years (SD=10.50), with Charcot neuropathy. Participants were provided with three types of shoes; prefabricated, relasting, and double rocker-modified shoes, each equipped with the same custom-made insole (CMI). Plantar pressure was recorded while walking on level ground, focusing on the forefoot, midfoot, and hindfoot. The study also investigated additional variables affecting plantar pressure distribution, including the pressure-time integral and contact area.

**FINDINGS:**

The type of shoe had distinct effects on the distribution of plantar pressure. The double rocker-modified shoe particularly impacted forefoot pressure during the terminal stance phase of the gait cycle. Peak plantar pressure at the forefoot increased by 5.37% with double rocker-modified shoes compared to relasting shoes. Both double rocker-modified and prefabricated shoes reduced midfoot peak plantar pressure by 8.73% and 11.97%, respectively. Similar trends were observed at the hindfoot, with reductions in peak plantar pressure. However, there were no significant differences in regional peak plantar pressure between the types of shoes except for the central forefoot (F (1.61, 22.5) = 5.69, p = 0.014).

**CONCLUSION:**

There were no significant differences in the effectiveness of prefabricated, relasting, and double rocker-modified shoes in reducing and redistributing peak plantar pressure in high-risk areas of chronic Charcot foot.

## INTRODUCTION

Charcot neuropathic osteoarthropathy, also known as Charcot foot, is a serious complication that can result from any disorder associated with sensory or autonomic neuropathy. This complication leads to bone destruction, deformities, and ulcerations that can lead to amputation if left untreated promptly and properly.^1^ Charcot foot is a major cause of morbidity and mortality in people with diabetes, with a 5-year mortality rate of 29% for those with Charcot foot alone and up to 56.6% for those who have undergone major amputations.^2^ Early diagnosis and treatment are crucial in preventing the progression of Charcot foot and minimizing the risk of complications. Charcot foot progresses through four stages: ***stage 0***, characterized by asymptomatic or nonspecific symptoms and normal radiographs; ***stage 1***, characterized by inflammation, swelling, and bone fragmentation visible on radiographs; ***stage 2***, features decreasing inflammation and evidence of healing on radiographs.; and ***stage 3***, noted for reduced swelling, joint stabilization, and new bone formation on radiographs. Each stage needs customized treatment approaches, emphasizing the importance of early recognition and appropriate management to prevent progression and complications.^3,4^ Treatment may involve immobilization, proper offloading devices, wound care, and surgery to repair or stabilize the affected foot.^2,5^

According to the 2017 Global Burden of Disease Research, the cost of caring for diabetic foot diseases and their complications in the United States was estimated to be around $237 billion.^2^ At present, there is no offloading device guideline for chronic Charcot foot without plantar ulcer. Proper therapeutic footwear is recommended to remove high pressure sites and in severe cases it also promotes the healing process for Charcot foot with ulcer.^6,7^

To prevent chronic Charcot foot ulceration, it is important to address gait dysfunction and biomechanical abnormalities that are common in people with diabetic neuropathic foot. These abnormalities often include an increase in the distribution of plantar pressure in sensitive areas. Notably, maintaining plantar pressure below the critical physiological skin tolerance level generally acknowledged to be less than 200 kPa to reduce the risk of developing foot ulceration and re-ulceration.^8^ The peak plantar pressure in Charcot foot mainly occurs in the midfoot area and redistribution of plantar foot pressure is considered the main method to reduce it. The footwear designs for redistributing midfoot peak plantar pressure are offloading pads, double rocker profile, proper shoe fitness, ankle-high, and knee-high offloading devices.^9,10^

Double rocker modification of diabetic shoes uses the rocker-shaped sole in the shoe to help redistribute plantar pressure. There was a suggestion that the double rocker modification may be effective in offloading the peak plantar pressure at the deformed midfoot area in people with chronic Charcot foot.^8,11^ Currently, there is very limited literature that focuses on evaluating the effect of double rocker modification on the midfoot and forefoot area of chronic Charcot foot without plantar ulcer.

The extra width of the Charcot foot can also be a problem for shoe fitting, as it may cause improper foot pressure loading during gait. To address this issue, a relasting technique may be used to reduce and redistribute peak plantar pressure. Relasting shoes are recommended for people with foot deformities, arthritis, or other conditions that make it difficult to find proper shoes that fit comfortably.^12^ The relasting technique requires reshaping the insole, outsole and heel of the shoe to better match the shape and size of the wearer's foot, which involves expanding the width of the shoe in the midfoot area to provide wider total contact of the plantar surface of the foot with the shoe.^13,14^ However, to the author’s knowledge, there is currently no research evaluating the effect of the relasting technique on midfoot peak plantar pressure of chronic Charcot foot without plantar foot ulcer. It was hypothesized that the relasting shoe with a custom-made insole (CMI) improves the offloading of the plantar aspect of the midfoot. Therefore, the aim of this study was to compare the effectiveness of prefabricated, relasting, and double rocker modified shoes to reduce and redistribute peak plantar pressure in the midfoot and forefoot in chronic Charcot without plantar ulcer. The outcome of this study may enhance the selection of suitable modified shoes to achieve optimal plantar pressure distribution, helping to prevent ulceration and re-ulceration in diabetic Charcot neuroarthropathy.

## METHODOLOGY

### Study population

Fifteen patients with Charcot neuroarthropathy were recruited from the Rehabilitation outpatient clinic, the Prosthetics and Orthotics clinic, the Diabetic clinic, and the Songklanagarind hospital inpatient department. Demographic characteristics including gender, body weight, height, shoe size, duration of diabetes, glycemic level, and foot-related problems were collected.

The type of foot was determined with the Foot Posture Index (FPI-6) by a clinician. FPI-6 is a clinical tool used to evaluate standing foot posture that provides a quantifiable measure, allowing clinicians to categorize foot types as supinated, neutral, or pronated. FPI-6 is widely recognized in clinical settings due to its reliability and ease of use, making it a valuable tool in both diagnostic and treatment planning processes for various foot-related conditions.^15,16^

This project was approved by the Research Ethics Committee (REC.62-421-11-1), Faculty of Medicine, Prince of Songkla University.

The inclusion criteria for all participants were an age range 18 to 80 years old, unable to feel the 10-gram monofilament, and able to walk at least 10 meters without any aid at a self-selected speed. Participants were excluded if they had an active infected plantar foot wound, severe peripheral vascular disease, angina, dyspnea, major or minor lower limb amputation, and unstable vital signs. All participants signed written informed consent forms after understanding the whole experimental protocol.

### Sample size determination

The sample size of 15 was determined to provide 80% probability of a clinically meaningful difference detection of 40 kPa in peak plantar pressure between footwear conditions. The standard deviation was set at 50 kPa and the alpha level was set at 0.05.^17^

### Shoe and custom-made insole fabrication

**Figure 1:**
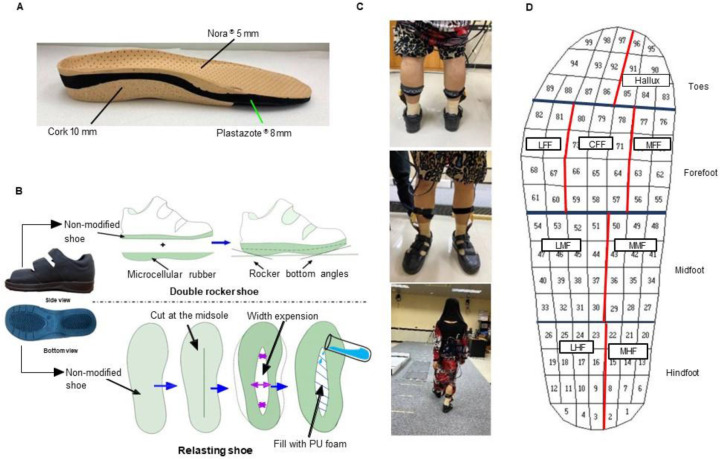
Charcot neuroarthropathic subjects are provided with a custom made insole (**A**), three types of shoes: prefabricated shoe; double rocker shoe, and relasting shoes (**B**), with the data collected during gait (**C**) from three main regions of foot as hindfoot, midfoot, forefoot. The forefoot is subdivided into four regions as follows: lateral forefoot (LFF), central forefoot (CFF), medial forefoot (MFF) and hallux using masking software. The medial and lateral division is performed for the midfoot and the hindfoot (**D**).

A certified orthotist fabricated custom-made insoles (CMI) from Nora^®^ 5 mm top layer, Plastazote^®^ 8 mm middle layer, and cork 10 mm base layer (**Figure 1-A**). All patients received CMI with three footwear conditions, prefabricated shoes, double rocker shoes, and relasting shoes. The design of the prefabricated shoes (CDM^*^: Cordoma International Co., Ltd.) are seamless lining with microcellular rubber outsole and a synthetic leather quarter. A certified orthotist modified the outsole of the prefabricated shoes with the addition of 10 mm ethylene vinyl acetate (EVA) 30 Shore A and reshaped it to make it a double rocker shoe with an angle of 10 degrees. Moreover, relasting shoes are fabricated by cutting the middle of the shoes and adding polyurethane foam to provide more room for the midfoot in the mediolateral direction as shown in **Figure 1-B**. A 5 mm thick layer of anti-slip microcellular rubber with 70 Shore hardness was added to both double rocker and relasting shoes, resisting wearing out and provide high stability.

### Evaluation of plantar pressure

The peak plantar pressure was collected during gait with the Pedar-X^®^ system (Novel Inc.; Munich, Germany), following the final fitting of the custom-made insole and three types of shoes. Data were collected from three gait cycles along a 10-meter walkway with self-selected speed. Before data collection, subjects walked along a 10-meter walkway at their comfortable speed to determine their walking speed and familiarize themselves with their shoes (**Figure 1-C**). The sensors were placed under CMI and each sensor was calibrated according to the guidelines. Novel Multimask software (Novel GmbH, Munich, Germany) was used to divide the foot area into 3 main regions, namely hindfoot, midfoot, and forefoot. The main three regions of the foot were divided into the medial hindfoot, lateral hindfoot, medial midfoot, lateral midfoot, medial forefoot, central forefoot, lateral forefoot, and big toe. Peak plantar pressure, pressure-time integral, contact area, and pressure mapping during gait were evaluated with three types of shoes. To evaluate the impact of shoes on plantar pressure distribution, each participant wore three types of shoes with the same CMI.

### Data analysis

The data's descriptive statistics were presented as mean and standard deviation. Statistical analysis was performed using Prism 5.0 (GraphPad Software, San Diego, CA, USA). Analyses were conducted on the affected foot. A repeated measures ANOVA was used to compare plantar pressure and derived parameters among prefabricated, double rocker, and relasting shoes to assess the impact of shoe modifications. Post hoc comparisons were conducted where significant differences were found. Statistical significance was set at p<0.05.

## RESULTS

### Participant characteristics

The participant demographics are shown in **Table 1**. Fifteen Charcot neuroarthropathic patients (6 (40%) males and 9 (60%) females) were included with a mean age of 60.73 (SD=10.5) years were included. The mean body mass index was 25.13 kg/m^2^ (SD=2.52). Charcot foot cases represented a long duration of diabetes (16.33 years (SD=6.05)) related to poor glycemic control, and the serum HbA1c level was 7.88% (SD=0.85). The results also showed that 66.67% of patients with Charcot arthropathy had a history of previous foot problems, such as foot ulcers or surgery. Patients with stage 3 Charcot arthropathy in this study showed a mean foot posture index (FPI-6) of 6.71 (SD=1.93).

**Table 1:** Demographic data of Charcot neuroarthropathic subjects.

Parameters	Mean	SD
**Age (y)**	60.73	10.50
**Body weight (kg)**	78.53	8.29
**Height (m)**	1.56	0.04
**BMI (kg/m^2^)**	25.13	2.52
**Shoe size (EU)**	40.40	1.25
**Duration of diabetes (y)**	16.33	6.05
**Serum HbA1C (% NGSP)**	7.88	0.85
**FPI-6**	6.71	1.93

### Regional peak plantar pressure

**Figure 2:**
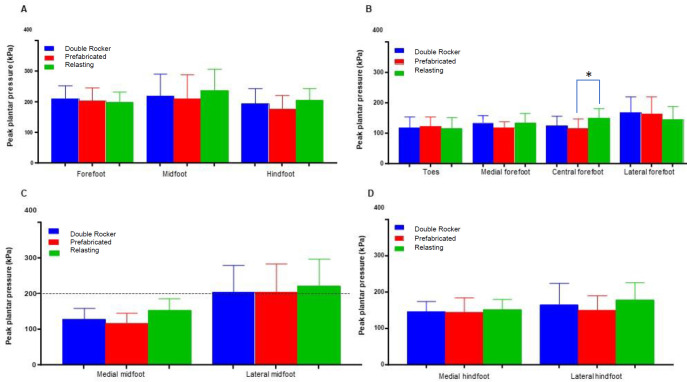
Peak plantar pressure during gait for three types of shoes—double rocker, prefabricated, and relasting—across different foot regions. The data is divided into: (A) Three main regions of the foot; (B) Four forefoot regions; (C) Two midfoot regions; (D) Two hindfoot regions. Significant differences are marked with an asterisk (*), indicating a p-value of less than 0.05.

The midfoot showed higher peak plantar pressure with three types of shoes compared to the forefoot and hindfoot regions of the foot. Moreover, the tendency for the peak plantar pressure was higher in the lateral midfoot compared to the medial midfoot, as shown in **Figure 2**.

The peak plantar pressure at the forefoot increased with double rocker-modified shoes compared to relasting shoes. Furthermore, the maximum plantar pressure was highest in the lateral forefoot with double rocker-modified shoes compared to relasting and prefabricated shoes. However, the central forefoot and medial forefoot peak plantar pressure is reduced with double rocker-modified shoes and prefabricated shoes (**Table 2**). There was no significant difference in regional peak plantar pressure among the measured variables except for the central forefoot with prefabricated and relasting shoes.

**Table 2:** Regional peak plantar pressure with three types of shoes PF: prefabricated; DR: double rocker and RL: relasting from different regions of the foot.

Foot region	Shoe type comparison	Mean diff.	p-value	Geisser-Greenhouse's epsilon	r^2^	F (DFn, DFd)
**Forefoot**	PF vs. DR	−6.60	0.609	0.818	0.047	0.69 (1.64, 22.90)
	RL vs. DR	−10.30	0.604			
	RL vs. PF	−3.65	0.910			
**Toes**	PF vs. DR	10.10	0.254	0.818	0.097	1.50 (1.64, 22.90)
	RL vs. DR	0.963	0.991			
	RL vs. PF	−9.09	0.219			
**Medial forefoot**	PF vs. DR	−9.99	0.391	0.860	0.100	1.56 (1.72, 24.10)
	RL vs. DR	2.86	0.894			
	RL vs. PF	12.90	0.352			
**Central forefoot**	PF vs. DR	−5.01	0.745	0.803	0.289	5.69 (1.61, 22.50)
	RL vs. DR	25.00	0.077			
	RL vs. PF	30.00	0.036^*^			
**Lateral forefoot**	PF vs. DR	−5.74	0.822	0.961	0.187	3.21 (1.92, 26.90)
	RL vs. DR	−23.80	0.101			
	RL vs. PF	−18.10	0.149			
**Midfoot**	PF vs. DR	−6.97	0.867	0.895	0.158	2.62 (1.79, 25.00)
	RL vs. DR	19.80	0.154			
	RL vs. PF	26.80	0.115			
**Medial midfoot**	PF vs. DR	−10.60	0.707	0.930	0.197	3.44 (1.86, 26.00)
	RL vs. DR	25.40	0.161			
	RL vs. PF	36.00	0.095			
**Lateral midfoot**	PF vs. DR	−1.09	0.995	0.945	0.116	1.83 (1.89, 26.50)
	RL vs. DR	17.60	0.296			
	RL vs. PF	18.70	0.162			
**Hind foot**	PF vs. DR	−16.50	0.666	0.748	0.099	1.55 (1.50, 21.00)
	RL vs. DR	11.10	0.564			
	RL vs. PF	27.60	0.253			
**Medial hindfoot**	PF vs. DR	−1.74	0.990	0.665	0.014	0.21 (1.33, 18.60)
	RL vs. DR	5.91	0.687			
	RL vs. PF	7.65	0.873			
**Lateral hindfoot**	PF vs. DR	−16.40	0.580	0.919	0.133	2.16 (1.84, 25.70)
	RL vs. DR	13.00	0.577			
	RL vs. PF	29.40	0.107			

### Regional pressure-time integral and contact area

The pressure-time integral and contact area from three regions of the foot are shown in **Table 3**. The pressure-time integral was highest at the midfoot compared to the forefoot and hindfoot using three types of shoes. The type of shoe has a minimum effect on the regional pressure-time integral. The overall contact area among the foot regions increased in the forefoot with all types of shoes followed by the midfoot and the hindfoot.

**Table 3:** Pressure-time integral and contact area with three types of shoes from three main regions of the foot during gait.

Foot region	Shoe type comparison	Mean diff.	p-value	Geisser-Greenhouse's epsilon	r^2^	F (DFn, DFd)
***Pressure-time integral***						
**Forefoot**	PF vs. DR	−6.96	0.301	0.866	0.140	2.28 (1.73, 24.30)
	RL vs. DR	−10.8	0.210			
	RL vs. PF	−3.86	0.698			
**Midfoot**	PF vs. DR	−2.94	0.919	0.947	0.037	0.54 (1.89, 26.50)
	RL vs. DR	5.18	0.764			
	RL vs. PF	8.12	0.634			
**Hind foot**	PF vs. DR	−9.46	0.493	0.678	0.101	1.58 (1.36, 19.00)
	RL vs. DR	2.87	0.767			
	RL vs. PF	12.30	0.357			
***Contact area***						
**Forefoot**	PF vs. DR	0.41	0.947	0.851	0.089	1.37 (1.70, 23.80)
	RL vs. DR	−1.43	0.513			
	RL vs. PF	−1.84	0.135			
**Midfoot**	PF vs. DR	2.29	0.275	0.971	0.159	2.64 (1.94, 27.20)
	RL vs. DR	−0.94	0.769			
	RL vs. PF	−3.22	0.132			
**Hind foot**	PF vs. DR	1.25	0.349	0.748	0.099	1.55 (1.50, 21.00)
	RL vs. DR	0.62	0.676			
	RL vs. PF	−0.63	0.675			

Note: PF: Prefabricated, DR: Double rocker, RL: Relasting

### Pressure mapping during gait

**Figure 3:**
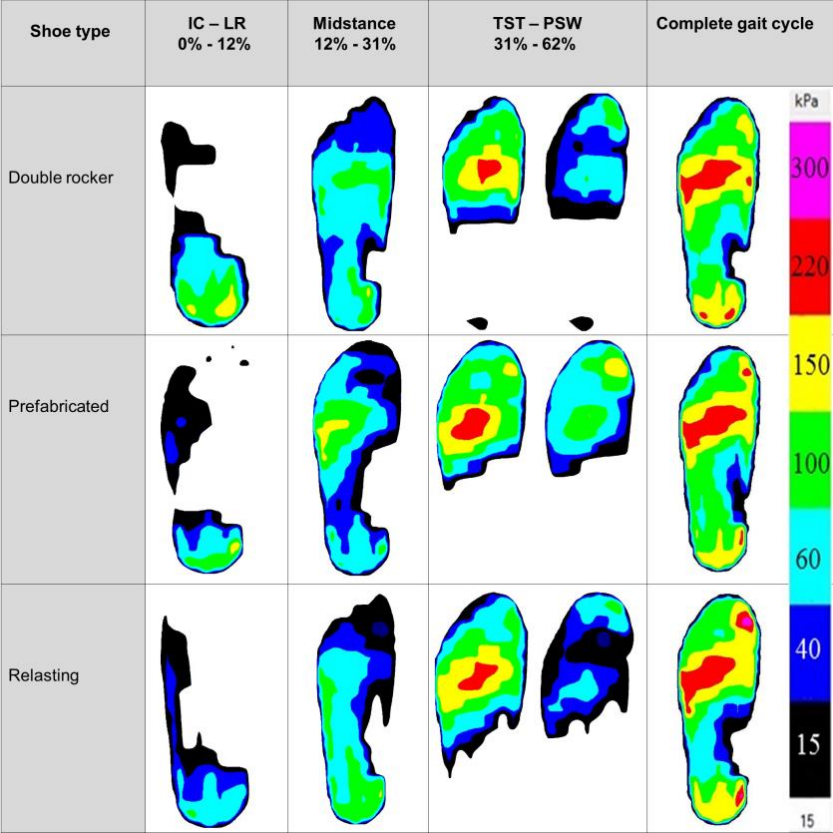
Pressure mapping during gait using double rocker, prefabricated, and relasting shoes. The data is shown for a single representative subject and includes: **IC – LR:** Initial Contact to Loading Response; **MST:** Midstance; **TST – PSW:** Terminal Stance to Pre-Swing.

The pressure distribution across different phases of the gait cycle with various types of shoes reveals distinct patterns (**Figure 3**). Relasting shoes exhibit reduced pressure from initial contact to loading response. Moreover, during midstance, a similar trend can be observed with relasting and double rocker shoes, but the pressure increased in the lateral forefoot with prefabricated shoes. During terminal stance, double rocker shoes promote a more centralized pressure distribution. Conversely, prefabricated and relasting shoes resulted in higher lateral foot pressure.

## DISCUSSION

This study found that relasting shoes increased the peak plantar pressure compared to prefabricated shoes and double rocker shoes at the central forefoot. However, opposite results were found in the lateral forefoot, as metatarsal heads are at high risk of ulceration and reulceration during gait. The peak plantar pressure was consistently less than 200 kPa with the modified shoes with custom-made insole at the medial midfoot and lateral midfoot, suggesting a potentially lower risk of ulceration in the midfoot region. These observations highlight the impact of shoe modifications on peak plantar pressure at specific regions, offering valuable insights into differential effects of various shoe types on foot pressure distribution.

Although the results did not reach statistical significance, they contribute to our understanding of how shoe modifications may affect plantar pressure distribution. The double rocker and prefabricated shoes demonstrated a reduction in peak plantar pressure in the midfoot and central forefoot areas compared to relasting shoes. This suggests a potential benefit in the offloading high-risk areas, which is critical to reduce the risk of ulceration.^18^

Shoe modification considering pathological foot biomechanics is one of the most common practices in normal clinical practice for Charcot neuroarthropathy.^19,20^ Shoe modifications, including rocker bottom outsoles, are effective in further reducing and redistribute peak plantar pressure in various foot complications.^21^ Rocker shoes provide a shorter contact time on heel strike and toe release, resulting in instability during gait in Charcot neuroarthropathy.^22^ However, relasting shoes that provide a wider mediolateral space for the foot to accommodate provide better stability during gait, but resulted in increased peak plantar pressure, especially at the forefoot and hallux compared to a double rocker shoe.^23^

Researchers investigated double rocker shoes in reduction of hindfoot and forefoot peak plantar pressure, while preventing excessive overloading at the midfoot. During gait, shoes with rocker sole peak plantar pressure decreased especially in the forefoot regions but increased in the midfoot.^24^ In this study, a similar pattern was observed in which there was a decrease in peak plantar pressure in both the forefoot and midfoot areas for patients with Charcot neuroarthropathy. The findings align with previous research that indicates that these areas are particularly vulnerable in the Charcot foot due to structural deformities and altered gait mechanics.^16,25^ The elevated pressure in these regions highlights the need for targeted interventions to better redistribute pressure during walking, particularly in the lateral midfoot and central to the medial forefoot.

Reducing peak plantar pressure while considering the importance of pressure-time integral to plantar ulceration, hence minimizing the combination of plantar pressure and the duration for which plantar pressure is applied to tissue, may be preferable than evaluating and reducing peak plantar pressure alone.^26^ In this study, shoe modification impacts both peak plantar pressure and pressure-time integral outcomes in the forefoot and midfoot regions. Furthermore, the contact area in different foot regions did not show statistically significant differences. This suggests that the choice of shoe type with CMI may not significantly affect the contact area in specific foot regions.^27^

There were several limitations while investigating the plantar pressure distribution among three types of therapeutic designs with CMI. The barefoot data was not included in this study due to the severity and potential to harm the plantar soft tissue during gait. The width dimension of the sensor insole could affect the accuracy of plantar pressure, especially in the midfoot, in Charcot neuroarthropathy. Only one type of rocker shoe was compared; however, other designs might be effective for populations facing severe foot complications. Further studies are required to evaluate the effectiveness of CMI and various shoe designs with varying rocker bottom sole angle and height on plantar pressure distribution in Charcot neuroarthropathy.

## CONCLUSION

There were nonsignificant trends of pressure distribution and offloading in the high-risk areas of the foot using different types of shoe with CMI. Prefabricated and double rocker shoes showed some effectiveness in reducing peak plantar pressure in the midfoot, and central forefoot, compared to relasting shoes. However, achieving significant offloading in areas of high risk, such as the lateral midfoot, remains a challenge, which requires further shoe modifications and consideration of the materials and design of CMI. These insights are crucial for improving footwear interventions for diabetic Charcot neuroarthropathy, with the aim of reducing the risk of ulceration while maintaining foot functionality.

## DECLARATION OF CONFLICTING INTERESTS

The authors declare no potential conflict of interest.

## AUTHORS CONTRIBUTION

**Muhammad Nouman:** Conceptualization, design, methodology, analysis, reviewing/revising manuscript, data interpretation. **Ravissada Apiputhanayut**: Conceptualization, design, methodology, analysis, investigation, writing original draft, data interpretation, ethic certification application.

**Tuanjit Narungsri:** Conceptualization, methodology, reviewing/revising manuscript.

**Suttipong Tipchatyotin:** Conceptualization, supervision, methodology, reviewing/revising manuscript.

**Tulaya Dissaneewate:** Conceptualization, supervision, methodology, reviewing/revising manuscript, final manuscript approval.

All authors have read and approved the final version of the manuscript.

## SOURCES OF SUPPORT

Faculty of Medicine, Prince of Songkla University, Songkhla Thailand.

## Footnotes

* CDM is the brand of the prefabricated shoe used in this study.
